# Synergistic effects of anlotinib and DDP on breast cancer: targeting the VEGF/JAK2/STAT3 axis

**DOI:** 10.3389/fphar.2024.1494265

**Published:** 2024-10-23

**Authors:** Hongmei Zhang, Chunling Liu, Ye Jin, Zheng Wang, Yi Guan, Zhenxian Jia, Tong Cui, Zhi Zhang, Xuemei Zhang

**Affiliations:** ^1^ Hebei Key Laboratory of Occupational Health and Safety for Coal Industry, School of Public Health, North China University of Science and Technology, Tangshan, Hebei, China; ^2^ School of Clinical Medicine, North China University of Science and Technology, Tangshan, Hebei, China; ^3^ Hebei Key Laboratory of Molecular Oncology, The Cancer Institute, Tangshan People’s Hospital, Tangshan, Hebei, China; ^4^ College of Life Science, North China University of Science and Technology, Tangshan, Hebei, China; ^5^ Affiliated Tangshan Gongren Hospital, Hebei Medical University, Shijiazhuang, Hebei, China

**Keywords:** anlotinib, apoptosis, autophagy, JAK2/STAT3, breast cancer

## Abstract

**Background:**

Anlotinib, a highly selective inhibitor of VEGFR2, has demonstrated significant anti-tumor effects in various cancers. However, its potential synergistic effects with DDP (cisplatin) in breast cancer (BRCA) remain to be fully elucidated. This study aims to discover the therapeutic efficacy of anlotinib on BRCA, specifically the synergistic effects with DDP, and to elucidate the underlying molecular mechanisms.

**Methods:**

BRCA cells were treated with anlotinib and/or DDP. The proliferation, migration and invasion capabilities of BRCA cells were evaluated using CCK-8 assays, cell cycle distribution, clone formation assays, wound healing assays and transwell assays. Cell apoptosis was detected by flow cytometry technique and Hoechst33342 fluorescence staining. The potential mechanism of anlotinib in the development of BRCA was predicted through bioinformatics analysis, and the mRNA or protein levels were subsequently quantified using qPCR, immunofuorescence and western blot. The anti-breast cancer efficacy of anlotinib was evaluated *in vivo* using a xenograft tumor model.

**Results:**

Our findings reveal that increased VEGFA expression in BRCA patients is associated with poorer prognosis, underscoring the need for targeted therapeutic strategies. We also demonstrate that both anlotinib and DDP independently inhibit BRCA cell growth, migration, and invasion, while their combination exhibits a synergistic effect, significantly enhancing the inhibition of these oncogenic processes. This synergy is further evident through the induction of apoptosis and autophagy in BRCA cells. Mechanistically, anlotinib’s effectiveness is linked to its inhibition of the JAK2/STAT3 pathway, a critical axis in BRCA progression. *In vivo* study further support these results, showing that anlotinib markedly inhibits tumor growth in xenografted mice.

**Conclusion:**

This study confirms the efficacy of anlotinib or in combination with DDP and elucidates the mechanism behind anlotinib’s effectiveness, highlighting its role in inhibiting the JAK2/STAT3 pathway.

## 1 Introduction

Breast cancer (BRCA) is the most prevalent cancer among women globally and remains the leading cause of cancer-related death in female population (Qiu et al., 2021; Siegel et al., 2023). Currently, surgery remains the most effective treatment for BRCA, complemented by adjuvant therapies including chemotherapy, radiotherapy, and targeted molecular therapy. These treatments are employed to eradicate residual cancer cells, inhibit cancer growth, reduce the recurrence, and enhance the long-term survival rate of patients with BRCA (Brady-West and McGrowder, 2011; Pasha and Turner, 2021). In clinical practice, a diverse array of chemotherapeutic drugs are utilized, among which cisplatin (DDP) continues to be a mainstay in the treatment of numerous malignant tumors, including BRCA (Yan et al., 2021; Li et al., 2023). However, despite its effective, DDP is susceptible to drug resistance and is associated with severe toxic side effects (Mehraj et al., 2021). Consequently, it is urgent to discover alternative therapeutic agent with superior curative effects, reduced side effects.

Anlotinib (AL3818) is a novel small molecule multi-targeted tyrosine kinase inhibitor, which targets vascular endothelial growth factor receptor (*VEGFR*), platelet derived growth factor receptor (*PDGFR*) and fibroblast growth factor receptor (*FGFR*) to comprehensively block tumor angiogenesis (Shen et al., 2018). It demonstrates a notable clinical efficacy in treating a variety of solid tumors, including BRCA, and is characterized by minimal adverse reactions (Ghozy et al., 2020; Hu et al., 2021). Previously, our laboratory has demonstrated that anlotinib combined with DDP significantly inhibited the proliferation of colorectal cancer (CRC) cells by antagonizing the *VEGFR/*Janus kinase 2 (*JAK2*)/signal transducer and activating transcription-3 (*STAT3*) (Jia et al., 2021). While the efficacy of anlotinib in treating breast cancer is well-established, the combined anti-tumor efficacy of Anlotinib and DDP in BRCA, as well as their underlying mechanism, remain to be fully elucidated.

Angiogenesis plays a crucial role in both tumorigenesis and distant metastasis (Huang et al., 2022; Mu et al., 2023). Vascular endothelial growth factor (*VEGF*) and its high-affinity receptor *VEGFR* are known to significantly enhance angiogenesis (Hicklin and Ellis, 2005). *VEGFA,* as the founding and most extensively studied member of the *VEGF* family, plays a pivotal role in tumor angiogenesis. The high expression of *VEGFA* leads to a worse prognosis in BRCA patients (Gong et al., 2021; Lu et al., 2021). During the progression and metastasis of malignant tumors, *STAT3* undergoes persistently activation in response to continuous growth signals. The *JAK2/STAT3* signal regulates various cytokines associated with this process, such as *VEGF* (Yang et al., 2016; Zhang et al., 2016). Recent studies have shown that autophagy can be induced by inhibiting the JAK2/STAT3 signaling pathway (Wang et al., 2018; Liang et al., 2019; Zhong et al., 2021).

In this study, we elucidate a novel mechanism underlying the synergistic anticancer effect of anlotinib combined with DDP in the treatment of BRCA by integrating bioinformatics predictions with validation through both *in vivo* and *in vitro* models. This research explores new applications of anlotinib and provide a crucial theoretical foundation for BRCA targeted therapy.

## 2 Materials and methods

### 2.1 Bioinformatics analysis

The Cancer Genome Atlas (TCGA) RNA-seq data and corresponding clinical data were downloaded from UCSC Xena platform (https://xena.ucsc.edu/) (Vivian et al., 2017). The differences in *VEGFA* mRNA expression between breast cancer and normal tissues were assessed using the Wilcoxon rank-sum test with a threshold of |log2FC| > 1 and *P* < 0.05. The difference in VEGFA protein level between breast cancer and normal tissues was evaluated using online Human Protein Atlas project (https://www.proteinatlas.org/) (Asplund et al., 2012).

Kaplan-Meier Plotter (http://kmplot.com) database was utilized to evaluate the prognostic value of *VEGFA* in BRCA (*FDR* < 0.05, *P* < 0.05) (Lánczky and Győrffy, 2021). Patients were stratified into high or low expression groups based on the median of *VEGFA* mRNA level. We assessed the impact of *VEGFA* on the overall survival (OS), relapse free survival (RFS), distant metastasis free survival (DMFS) and post-progression survival (PPS) of breast cancer patients by calculating hazard ratio (*HR*) and 95% confidence interval (*95% CI*).

### 2.2 Cell culture and drug treatment

Human breast cancer cells (MCF-7 and MDA-MB-231) were purchased from Procell (Wu Han, China). Cells were cultured in DMEM medium (Thermo Fisher Scientific, Waltham, MA, United States) containing 10% fetal bovine serum (FBS; Thermo Fisher Scientific, Waltham, United States) and 1% antibiotics (Solarbio, Beijing, China) in the presence of 5% CO_2_ at 37°C. The antibiotics were added at a final concentration of 100 U/mL of penicillin and 100 μg/mL of streptomycin. All cell lines were used between passages 3 to 10 to maintain consistency in cellular characteristics.

Anlotinib was a gift from Chia Tai Tianqing Pharmaceutical Company (Nanjing, China). DDP was purchased from Haosen (Lianyungang, China). Anlotinib was dissolved in dimethyl sulfoxide (DMSO; Sigma, St. Louis, United States) and stored at −20°C shielded from light. BRCA cells were treated with anlotinib or DDP at concentrations of 0, 5, 10, 20 and 40 μM. The combined treatment group was administered 10 μM anlotinib and 8 μM DDP. For all *in vitro* experiments, triplicate wells were used to ensure the reliability and reproducibility of the results.

### 2.3 Cell proliferation assay

The effects of anlotinib and DDP on the proliferation of BRCA cells were detected by CCK-8 assay. MCF-7 or MDA-MB-231 cells were seeded at a density of 5 × 10^3^ cells/well in 96 well plates and cultured overnight, and then treated with or without anlotinib and DDP. CCK-8 (Dojindo, Kumamoto, Japan) was then added at the designated time. After 2 h at dark, the optical density (OD) at 450 nm was measured with a microplate reader (Tecan, Männedorf, Switzerland).

### 2.4 Clone formation assay

After treated with or without anlotinib and/or DDP, 1 × 10^3^ breast cancer cells were then seeded in a 60 mm dish and cultured for 14 days. After fixation with 4% paraformaldehyde and staining with 0.1% crystal violet, colonies (≥ 50 cells) were counted under an IX71 inverted microscope (Olympus, Tokyo, Japan).

### 2.5 Wound healing assay

Cell migration was measured using a wounding healing assay. Upon reaching 90% confluence, the cell monolayer was scratched using a 200 μL pipette tip, creating a linear wound perpendicular to the culture plate. Subsequently, cells were incubated in a medium containing anlotinib and/or DDP for 48 h. Photographs were taken with an IX71 inverted microscope at 0h, 24h and 48 h time point and the wound areas were measured by Image J.

### 2.6 Cell migration and invasion assays

BRCA cells treated with anlotinib and/or DDP were resuspended in serum-free DMEM medium. Matrigel coated or uncoated transwell chambers (Corning, Corning, NY, United States) were seeded with cell suspensions (1 × 10^5^ cells/200 μL) and cultured for 24 h or 48 h in DMEM medium with 20% FBS. Non-migrated cells were scraped off with a cotton swab. Cells in the bottom wall of chambers were fixed with 4% paraformaldehyde and stained with 0.1% crystal violet, and then the migrated or invaded cells were photographed under an inverted microscope.

### 2.7 Flow cytometry analysis for cell cycle distribution

BRCA cells (MCF-7 and MDA-MB-231) were treated with anlotinib (10 µM) for 24 h, while control groups received DMSO. After treatment, cells were harvested, fixed in 70% ethanol at −20°C for 2 h, and then washed with PBS. Cells were stained with propidium iodide (50 μg/mL) and RNase A (100 μg/mL) in PBS, incubating at 37°C for 30 min in the dark. DNA content was measured by flow cytometry (Beckman Coulter, Brea, United States).

### 2.8 Apoptosis assays

Hoechst 33,324 staining was used to observe nuclear morphology. After treated with anlotinib and/or DDP, BRCA cells were fixed with 4% paraformaldehyde, and then stained with Hoechst33324 (Solarbio, Beijing, China). Cell size and nuclear morphology were observed using IX71 fluorescence microscope (Olympus, Tokyo, Japan).

We used Annexin V-PE/7-AAD kit (BD Biosciences, San Jose, CA, United States) to evaluate cell apoptosis. According to the manufacturer’s protocol, cells were stained with 5 μL Annexin V-PE and 5 μL 7-AAD working solution for 15min in the dark. Flow cytometric analysis was then performed using a flow cytometer (Beckman Coulter, Brea, United States).

### 2.9 RNA extraction, cDNA synthesis, and qPCR

Total RNA was extracted using Trizol reagent (Invitrogen, CA, United States), and then reversed transcribed into cDNA using the RevertAid First Strand cDNA Synthesis Kit (Thermo Fisher Scientific, United States). The qPCR was conducted using the ABIPRISM^®^ 7900HT fast real-time PCR system (Applied Biosystems, Foster City, United States). For each gene, 2^−ΔΔCT^ was calculated to determine its relative expression (Amuthalakshmi et al., 2022). Primers were listed in Supplementary Table S1.

### 2.10 Immunofuorescence analysis

The cells were inoculated on the cover slide at a density of 2 × 10^4^ cells/well and incubated at 37°C overnight. The cells were fixed with 4% paraformaldehyde at room temperature for 15 min and closed with 10% goat serum for 30 min. Anti-BAX, anti-BCL2, anti-P62 (Affinity, Jiangsu, China) antibodies were incubated at 4°C overnight. Subsequently, the cover slides were incubated with FITC-conjugated Goat Anti-Rabbit IgG (H + L) antibodies (Affinity, Jiangsu, China) at room temperature for 1 h. The specimens were then stained with DAPI. The stained samples were observed and analyzed using a fluorescence microscope (Zeiss, Germany).

### 2.11 Western blot analysis

MCF-7 or MDA-MB-231 cells were lysed using RIPA lysis buffer, and the protein concentrations were subsequently determined. Protein samples with equal mass were subjected to separation using 6%, 10% or 12% SDS polyacrylamide gel electrophoresis and then transferred onto polyvinylidene fluoride (PVDF) membranes. The membranes were incubated with the indicated primary antibodies and then the corresponding horseradish peroxidase (HRP) conjugated secondary antibodies. Proteins were finally visualized with enhanced chemiluminescence (ECL) luminescence reagents (Amersham, United Kingdom). β-actin served as a reference control. Anti-β-actin, anti-phospho-VEGFR2 and anti-LC3B were purchased from Cell Signaling Technology (CST, Massachusetts, United States). Antibodies (anti-BAX, anti-BCL2, anti-PARP1, anti-SQSTM1/p62, anti-JAK2, anti-STAT3, anti-phospho-JAK2 and anti-phospho-STAT3) were obtained from Abcam (Eugene, United States).

### 2.12 Xenograft mouse model

Female BALB/c-nu mice (4 weeks of age) were purchased from Beijing Huafukang Biotechnology Co., LTD. After 1 week of adaptive feeding, the mice received a subcutaneous injection of 3 × 10^6^ MDA-MB-231 cells into the right armpit. The tumor volumes were calculated according to the formula: length × width^2^ × 0.5. When the tumor volumes reached about 100 mm^3^, the mice were randomly divided into control group (sterile PBS loaded with drugs) and anlotinib treatment group (5 mg/kg) (Zhu et al., 2022). The mice were then given PBS or anlotinib intragastric treatment daily for 14 days. Tumor volumes and the mice body weights were recorded every 2 days. After 14 days of treatment, the mice were euthanized via cervical dislocation. The subcutaneous tumor tissue was excised, photographed and subjected to staining. The animal experiment was approved by the Experimental Animal Ethics Committee of North China University of Science and Technology (2023-SY-016).

### 2.13 Statistical analysis

GraphPad Prism 8 software was used to draw statistical graphs. One-way ANOVA and Student’s t-test were used to analyze the statistical significance of the measured variables. *P* < 0.05 was considered statistically significant.

## 3 Results

### 3.1 Increased VEGFA expression in BRCA patients associated with adverse prognosis

**FIGURE 1:**
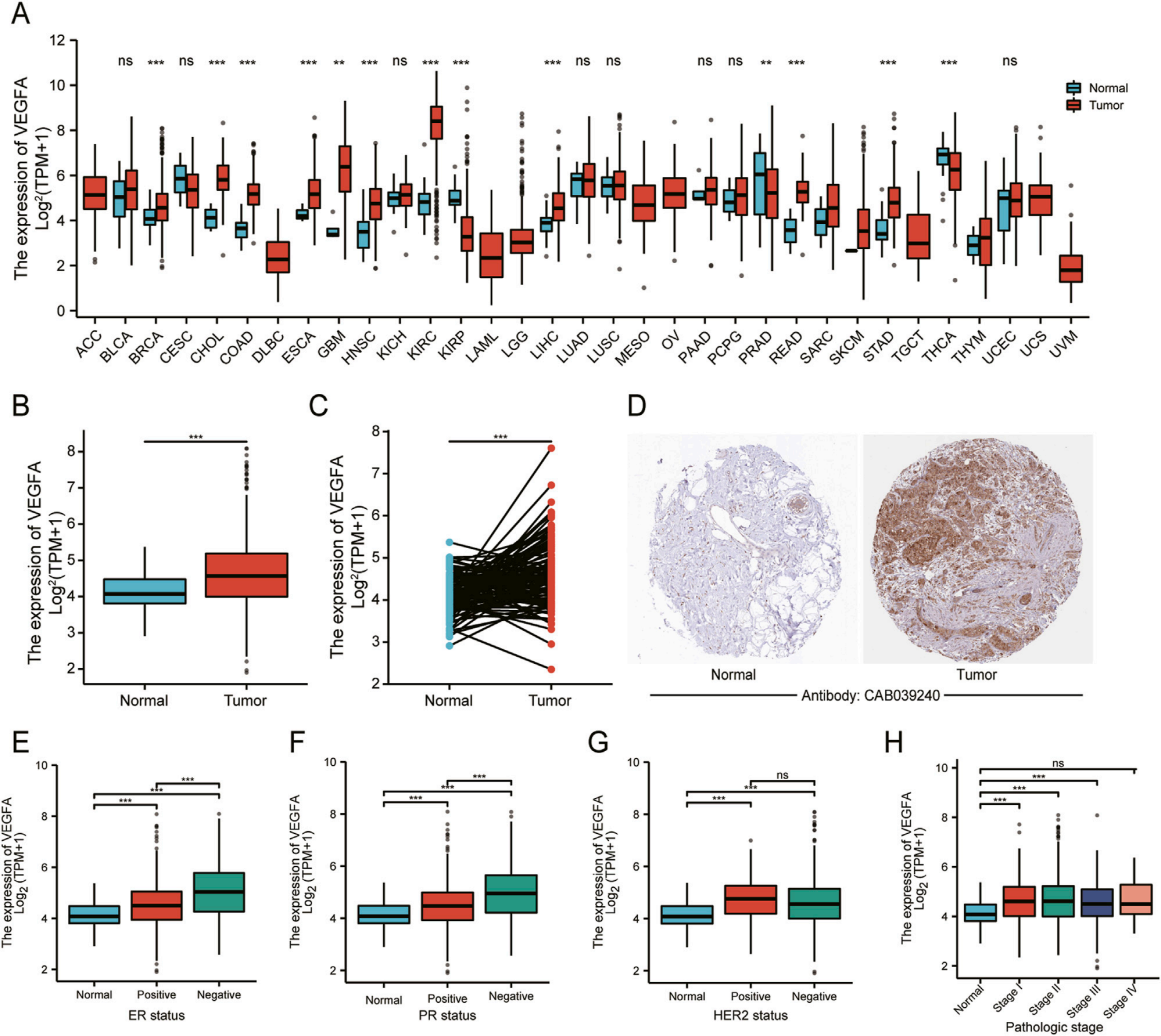
VEGFA expression and its relationship with different clinicopathological features in BRCA. **(A)** Pan-cancer analysis of *VEGFA* expression in tumor and adjacent normal tissues. *VEGFA* expression in **(B)** unpaired and **(C)** paired tumor and normal tissues. **(D)** Immunohistochemistry analysis of VEGFA protein. VEGFA expression in relation to **(E)** ER status, **(F)** PR status, **(G)** HER status and **(H)** pathologic stage. Data shown as mean ± SD. **P < 0.01, ***P < 0.001, ns: no significance.

In this study, we analyzed TCGA transcriptome data to evaluate *VEGFA* expression across various cancers and normal tissues. The results revealed that *VEGFA* was up-regulated in several cancer types, including BRCA, but downregulated in KIRP, PRAD and THCA (Figure 1A). Additionally, *VEGFA* transcript levels were higher in both unpaired and paired samples from TCGA-BRCA compared to normal tissues (Figures 1B, C). We also found that VEGFA protein expression was significantly elevated in BRCA tissues compared to normal tissues (Figure 1D).

Next, we evaluated *VEGFA* expression in different subgroups. In relation to ER, PR and HER2, we found that *VEGFA* expression was significantly higher in ER^−^ and PR^−^ breast cancer tissues compared to ER^+^ and PR^+^ tissues (Figures 1E, F). However, there was no significant difference of *VEGFA* between HER2^−^ and HER2^+^ tissues (Figure 1G). According to the TNM staging system, our data showed a significant increase in *VEGFA* expression in patients at stage Ⅰ, Ⅱ and Ⅲ, but not at stage Ⅳ (Figure 1H), which suggests that the elevated expression of VEGFA is prominent in the earlier stages of BRCA but not in the advanced stage.

**FIGURE 2:**
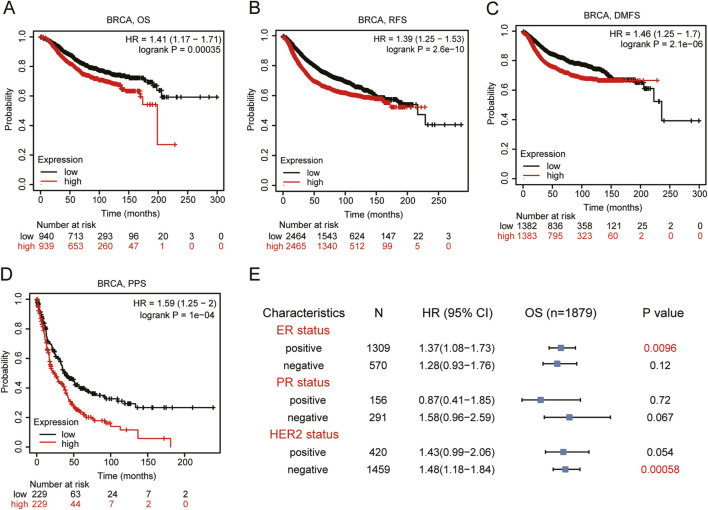
Prognosis analysis of VEGFA expression in BRCA patients. Kaplan-Meier plotter of **(A)** Overall survival, **(B)** relapse free survival, **(C)** distant metastasis free survival, and **(D)** post-progression survival, **(E)** Forest plot between VEGFA expression and overall survival categorized by different ER, PR, HER2 status.

Based on the data from Kaplan-Meier Plotter database, we found a strong association between higher *VEGFA* expression and the poor prognosis in BRCA patients (Figures 2A–D) with HR (95% CI) for OS, RFS and PPS of 1.41 (1.17–1.71), 1.39(1.25–1.53) and 1.59 (1.25–2.00), respectively. We further analyzed the relationship between *VEGFA* expression and OS of BRCA patients in different subgroups. The results indicated that (Figure 2E) high *VEGFA* expression correlated with poor prognosis in both ER^+^ (*HR* = 1.37, *95% CI* = 1.08–1.73, *P* = 0.0096) and HER2^−^ (*HR* = 1.48, *95% CI* = 1.18–1.84, *P* = 0.00058) BRCA patients. However, no significant correlation was found between *VEGFA* expression and OS, regardless of the PR status. These findings suggest that high VEGFA expression, when associated with specific clinicopathological features, correlates with the poor prognosis in BRCA patients.

### 3.2 Anlotinib inhibit BRCA cells growth *in vitro* and *in vivo*

**FIGURE 3:**
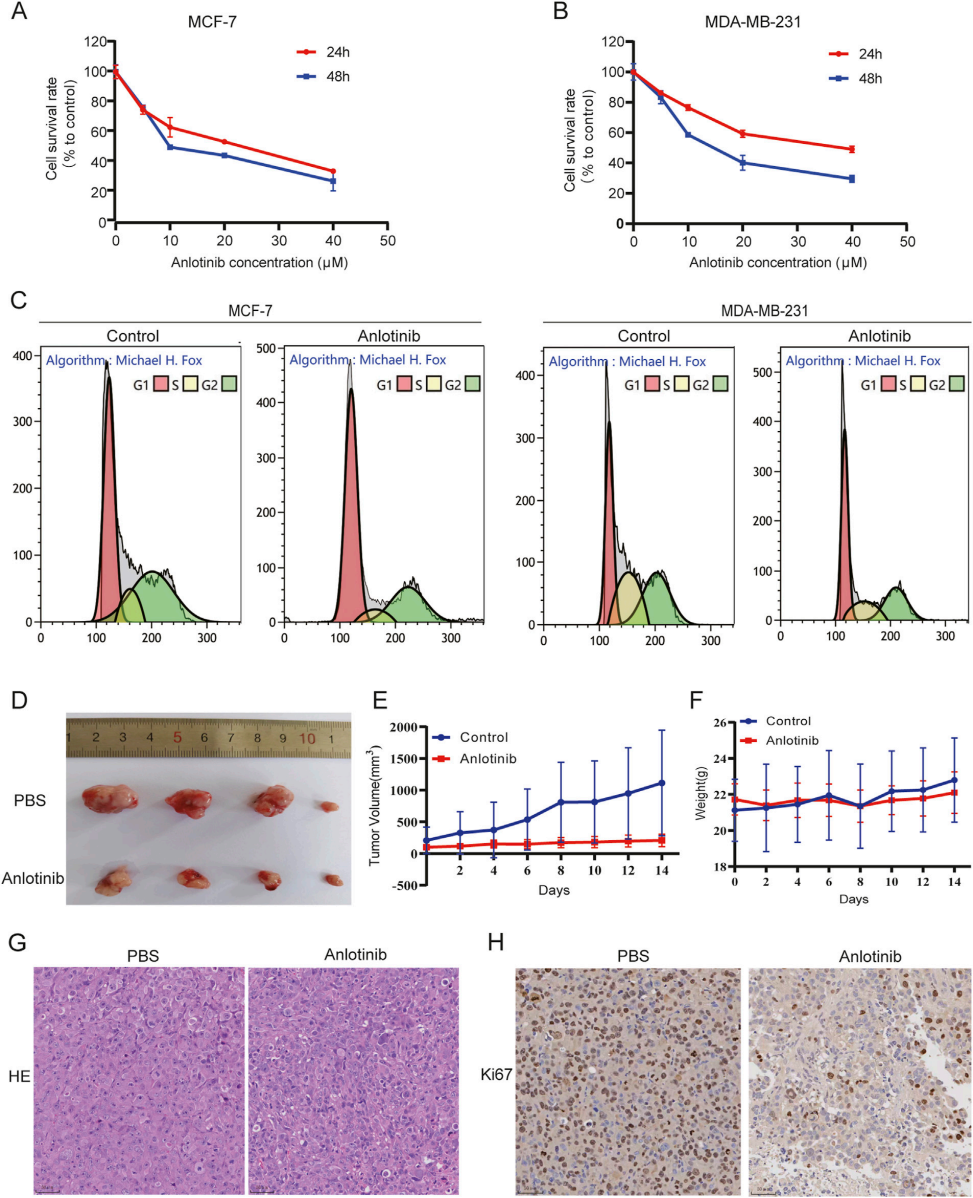
Anlotinib inhibit BRCA cells growth. Cell proliferation analysis by CCK8 assays in **(A)** MCF7 and **(B)** MDA-MB231 cells. **(C)** Effect of anlotinib on BRCA cell cycle distribution. **(D)** Subcutaneous tumor volumes comparison in nude treated with or without anlotinib. Tumor growth curves depicting **(E)** the volume and **(F)** weight of nude mice. Representative immunohistochemical image showcasing **(G)** HE stain and **(H)** Ki-67 in nude mice.

To evaluate the impact of anlotinib on BRCA cell proliferation, we performed CCK-8 assays using MCF-7 and MDA-MB-231 cells. The results indicated that anlotinib significantly inhibited cell viability in a dose- and time-dependent manner (Figures 3A, B). We also performed flow cytometry analysis to assess cell cycle distribution. The results indicate that treatment with anlotinib leads to a significant increase in the percentage of cells in the G1 phase and a corresponding reduction in the G2/M phase, compared to the control group (Figure 3C; Supplementary Figure S1), which supporting the observed inhibition is primarily due to reduced cell proliferation. To evaluate the *in vivo* therapeutic effect of anlotinib on BRCA, we established a mouse model of tumor transplantation by subcutaneous injection of MDA-MB-231 cells into the armpits of nude mice. Compared to the control group, anlotinib significantly inhibited tumor growth, with no weight loss (Figures 3D–F). HE stain and Ki-67 immunohistochemistry were used to evaluate cell proliferation. The results showed more nuclear mitotic images in control group than in anlotinib group, and the expression of Ki-67 was significantly reduced in the anlotinib-treated group (Figures 3G, H). These findings suggested that anlotinib had a strong anti-tumor effect both *in vivo* and *in vitro*.

### 3.3 Combined effects of anlotinib and DDP on cell proliferation and migration of BRCA

**FIGURE 4:**
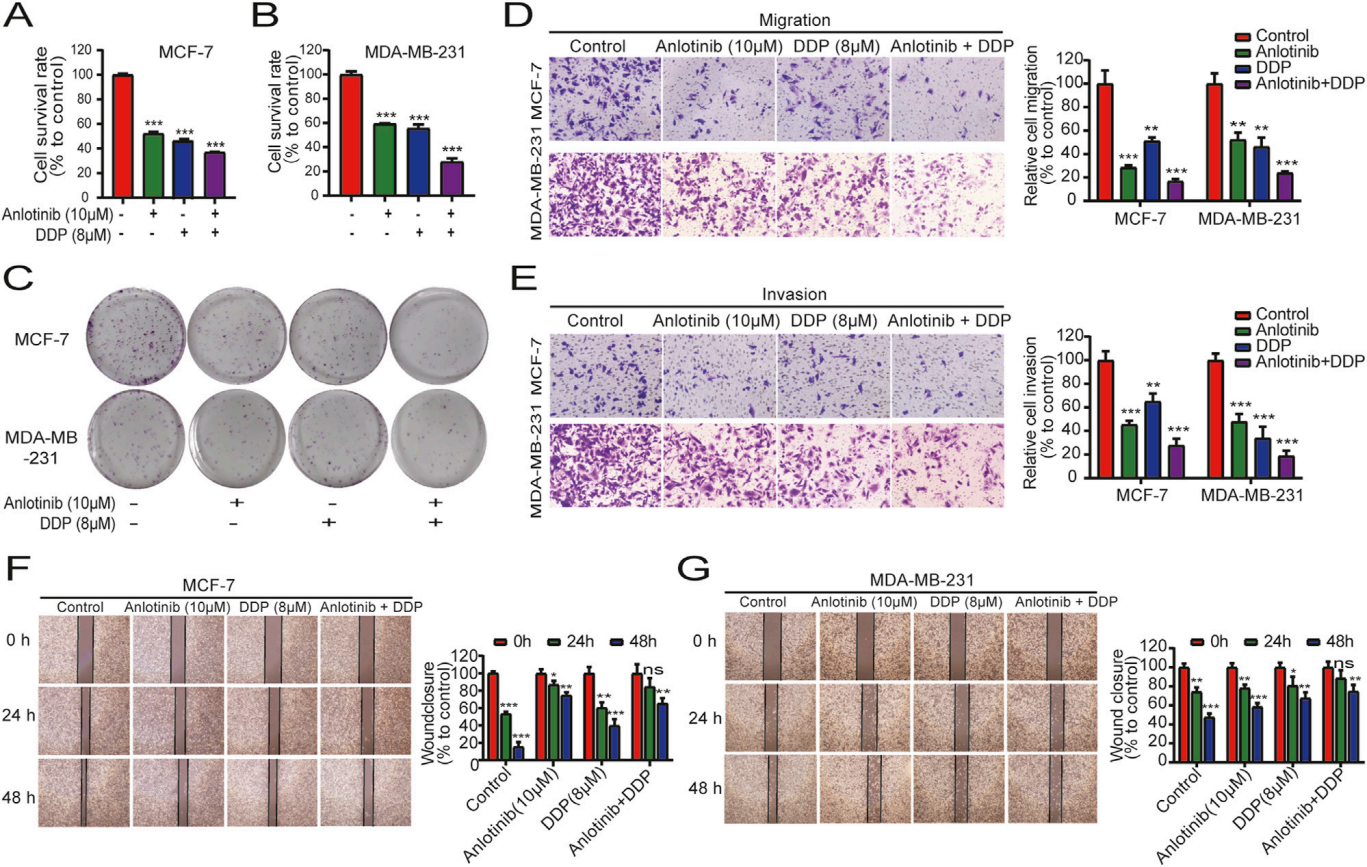
Combined effects of anlotinib and DDP on cell proliferation and migration of BRCA. The proliferation analysis by **(A)** CCK8 assays **(A, B)** and colony formation assays **(C)**. Transwell assays examined the **(D)** migration and **(E)** invasion capabilities of BRCA cells (magnification: ×200). **(F, G)** Wound healing assays illustrated the migration of BRCA cells (magnification: × 40). Data shown as mean ± SD. **P < 0.01, ***p < 0.001.

To determine the combined impact of anlotinib and DDP on breast cancer cell proliferation, we conducted CCK-8 assays using MCF-7 and MDA-MB-231 cells. When used in combination, anlotinib and DDP exhibited a synergistic effect, significantly enhancing the inhibition of breast cancer cell proliferation (Figures 4A, B). Colony formation assays further corroborated this synergistic effect, demonstrating a greater reduction in the number of cell clones in the combination treatment group compared to the single-agent treatment groups (Figure 4C). Next, we performed transwell assays to evaluate the effects of the combination of anlotinib and DDP on the migration and invasion of BRCA cells. The results showed that both anlotinib and DDP significantly inhibited cell migration and invasion, with the combination treatment group showing the most pronounced inhibition (Figures 4D, E). Additionally, wound-healing assays also revealed a notable delay in wound closure under the influence of anlotinib and/or DDP (Figures 4F, G). These findings suggest that anlotinib and DDP, particularly when used in combination, effectively inhibit the proliferation and migration of breast cancer cells.

### 3.4 Synergistic effects of anlotinib and DDP on apoptosis and autophagy in BRCA cells

**FIGURE 5:**
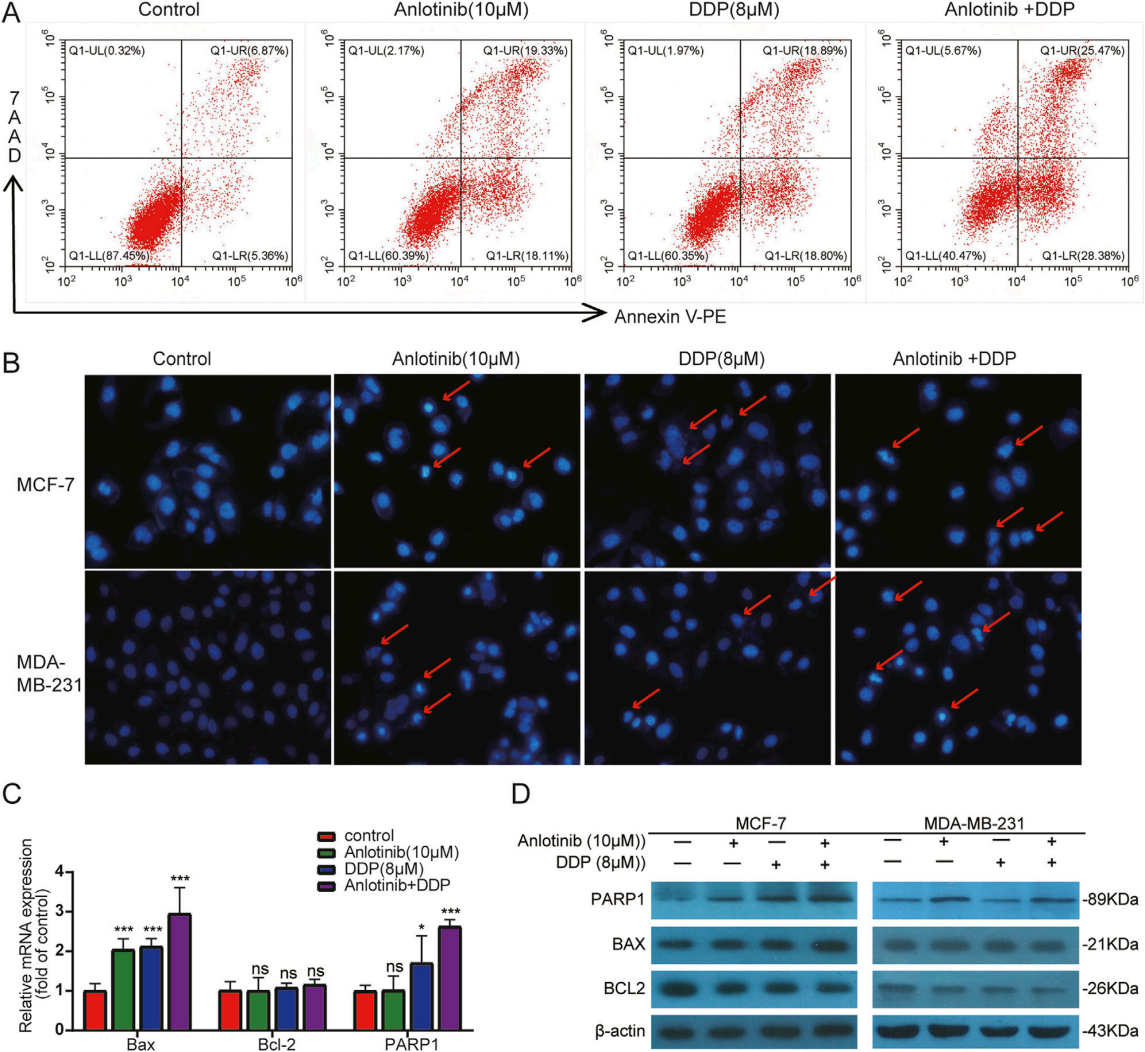
Apoptosis detection in BRCA cells by combined treatment with anlotinib and DDP. **(A)** Flow cytometry analysis. **(B)** Hoechst33324 staining. Apoptotic bodies are marked with red arrows (magnification: ×200). **(C)** The mRNA levels of apoptosis-related genes. **(D)** Apoptosis-related proteins (BAX, BCL2, PARP1) detection by western blot. Data shown as mean ± SD. *p < 0.05, **p < 0.01, ***p < 0.001, ns: no significance.

**FIGURE 6:**
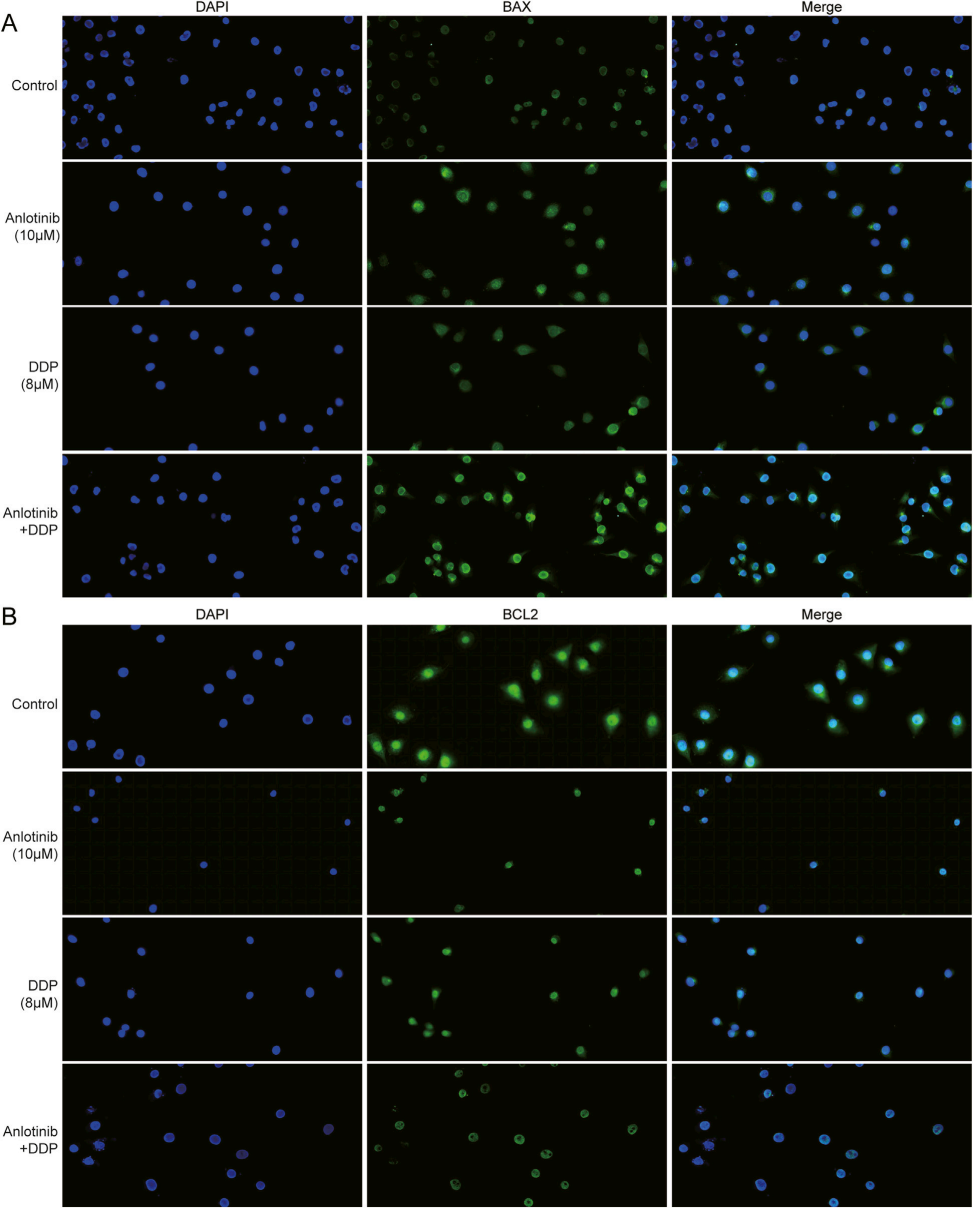
Immunofluorescence assay of apoptosis proteins in BRCA cells treated with anlotinib and/or DDP. The fluorescence intensity of **(A)** BAX and **(B)** BCL2 (magnification: ×200).

We employed flow cytometry and Hoechst33324 staining to evaluate the impact of anlotinib and DDP on the apoptosis of breast cancer cells. Flow cytometry analysis revealed that anlotinib and/or DDP significantly induced both early and late apoptosis of MCF-7 cells (Figure 5A). In BRCA cells treated with anlotinib and DDP, notable changes in nuclear morphology were observed (Figure 5B). Additionally, the qPCR results showed that the combination of anlotinib and DDP significantly upregulated the expression of *BAX*, *PARP1*; while no significant difference was observed in *BCL2* expression (Figure 5C). Western blot analysis revealed a marked increase in BAX and PARP1 expression in cancer cells treated with anlotinib alone or in combination with DDP, accompanied by a decrease in BCL2 expression (Figure 5D). Immunofluorescence analysis corroborated these findings, demonstrating a significantly increase in BAX fluorescence intensity in cancer cells treated with anlotinib alone or in combination with DDP, while the fluorescence intensity of BCL2 was decreased (Figures 6A, B). These results indicate that anlotinib promotes apoptosis by upregulating pro-apoptotic genes and inhibiting anti-apoptotic genes.

**FIGURE 7:**
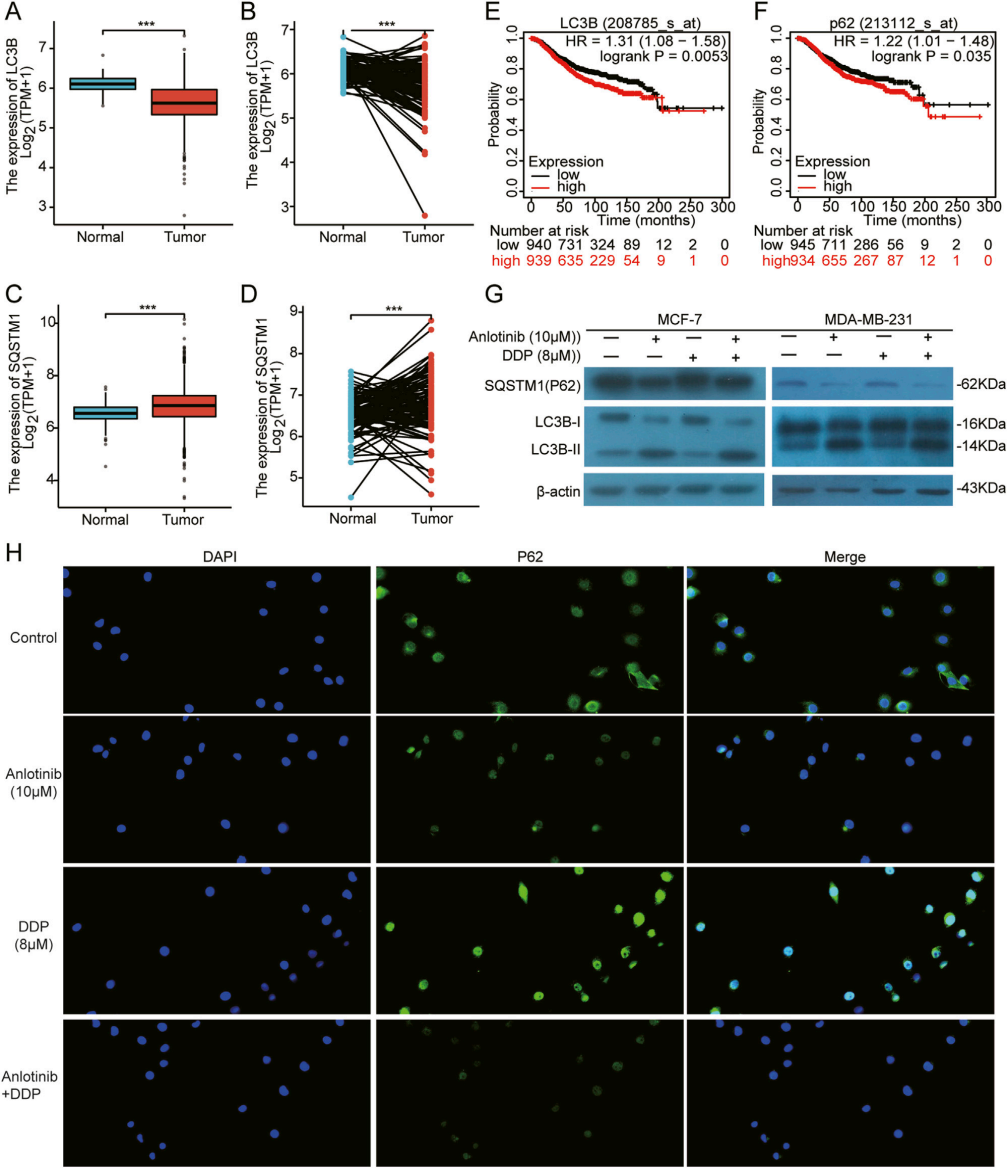
Induction of autophagy in BRCA cells by Anlotinib combined with DDP. **(A–D)** The expression of autophagy markers *LC3B* and *SQSTM1/P62* in both unpaired and paired samples. Kaplan Meier Plotter of overall survival by **(E)** *LC3B* and **(F)** *SQSTM1/P62* expression in BRCA patients. **(G)** Detection of key autophagy proteins (LC3B Ⅱ/Ⅰ, SQSTM1/P62) in BRCA cells. **(H)** The fluorescence intensity of P62 (magnification: ×200). Data shown as mean ± SD. ***p < 0.001.

Autophagy and apoptosis are crucial cellular processes that maintain homeostasis. The excessive autophagy can lead to cancer cell death through apoptosis (Tsujimoto and Shimizu, 2005). Therefore, we analyzed the expression of autophagy-regulating genes *LC3B* and *SQSTM1/P62*. Based on TCGA breast cancer data, there was a demonstrated low expression of *LC3B* and a high expression of *P62* in BRCA tissues compared to normal tissues (Figures 7A–D). Additionally, higher expression of *LC3B* and *P62* correlated with the poor prognosis for BRCA patients (Figures 7E, F). More importantly, through the immunofluorescence and western blot assays, we observed increased LC3B-Ⅱ and decreased P62 in MCF-7 and MDA-MB-231 cells co-treated with anlotinib and DDP (Figures 7G, H). These results suggest that anlotinib induces autophagy, contributing to the inhibition of breast cancer cell proliferation.

### 3.5 Anlotinib enhances BRCA treatment by targeting the JAK2/STAT3 pathway

**FIGURE 8:**
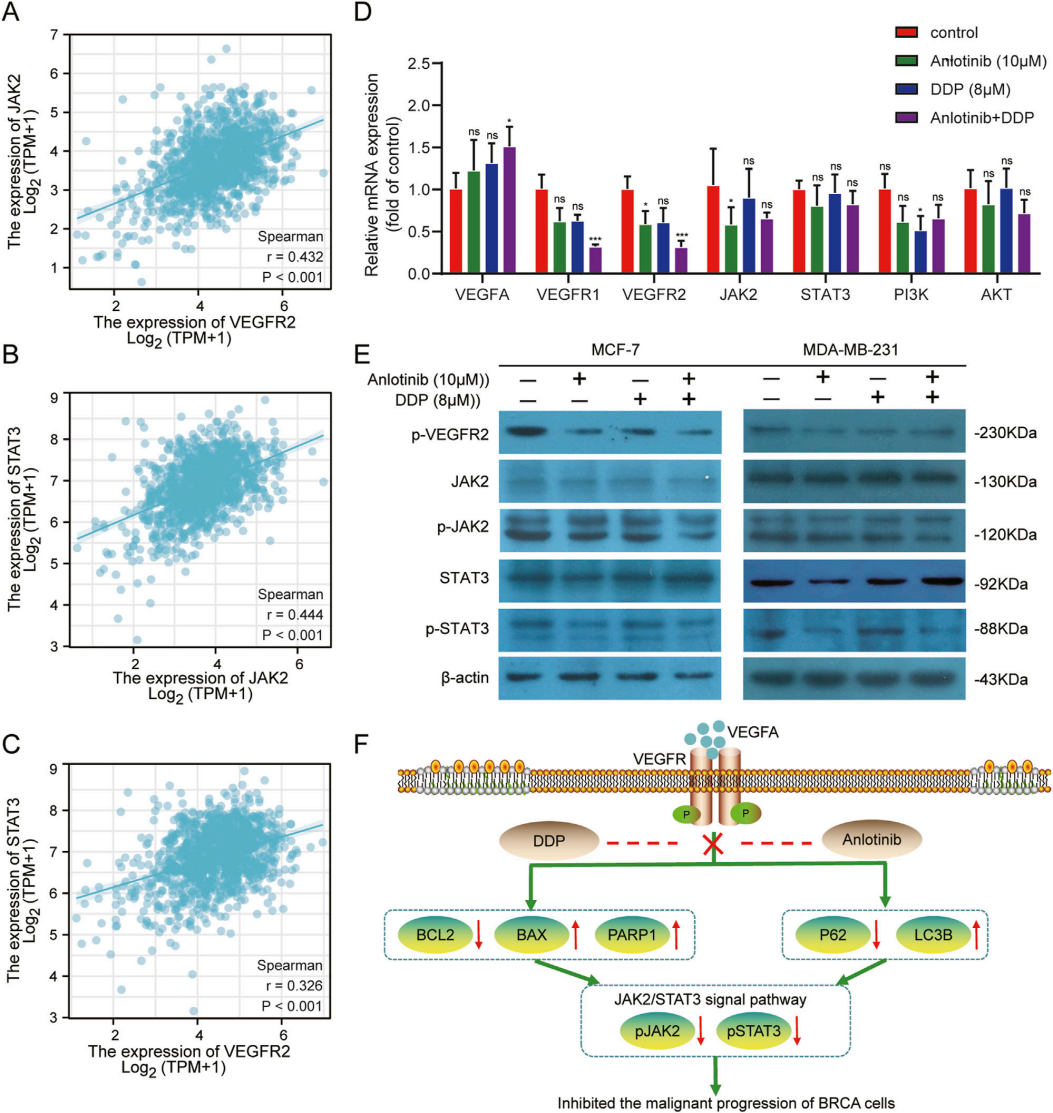
Inhibition of the VEGF/JAK2/STAT3 signaling pathway by combined Anlotinib and DDP treatment. **(A–C)** The correlation analysis among VEGFR2, JAK2 and STAT3. **(D)** Evaluation of mRNA levels of core genes in the VEGF/JAK2/STAT3, PI3K/AKT pathway in BRCA cells. **(E)** Assessment of protein expression in VEGFR/JAK2/STAT3 signaling pathway. **(F)** The mechanism by which anlotinib combined with DDP played its anticancer effect in BRCA. Data shown as mean ± SD. *p < 0.05, **p < 0.01, ***p < 0.001, *ns*: no significance.

Bioinformatic analyses suggested a potential interaction between VEGFR2 and JAK2/STAT3 pathway (Figures 8A–C). Anlotinib, a highly selected small molecule inhibitor of *VEGFR2*, was evaluated for its effects on target gene expression using qPCR. The results showed that the combined of anlotinib and DDP significantly reduced the expression of *VEGFR1* and *VEGFR2*, while increasing *VEGFA* expression. There was no significant difference in the mRNA levels of *JAK2*, *STAT3* and *PI3K/AKT*. Interestingly, in MCF-7 treated solely with anlotinib, *JAK2* expression was lower than that in the control group (Figure 8D *; P* < *0.05)*. Western blot analysis further confirmed the interaction between VEGFR2 and the JAK2/STAT3 pathway. Treatment with anlotinib and DDP not only reduced the expression of VEGFR2, but also inhibited the phosphorylation of JAK2 and STAT3, without affecting their total protein levels (Figure 8E). These findings suggest that anlotinib inhibit the phosphorylation of *JAK2/STAT3* pathway in BRCA cells, thereby impacting cancer progression.

## 4 Discussion

With the in-depth study of the pathogenesis of cancer and the continuous development of precision medicine, the treatment approaches of BRCA are constantly improved. Currently, platinum-based systemic chemotherapy remains the primary treatment for patients with advanced recurrent and metastatic breast cancer; however, targeted drugs have emerged as an effective supplement to neoadjuvant therapy (Bianchini et al., 2021). Combining targeted drugs with chemotherapeutic agents can potentially overcome the resistance that single chemotherapeutic drugs often encountered, offering new treatment stategies for BRCA (Jaaks et al., 2022).

Anlotinib is a third-line, broad-spectrum anticancer drug that has been shown in clinical trials to improve the efficacy of a variety of malignancies, including breast cancer (Hu et al., 2021), non-small cell lung cance (Han et al., 2018), thyroid cancer (Ruan et al., 2019), and intrahepatic cholangiocarcinoma (Song et al., 2020). Recent preclinical study has demonstrated synergistic cytotoxic effects of anlotinib in combination with the chemotherapeutic drug 5-FU against small cell lung cancer, both *in vitro* and *in vivo* (Xia et al., 2022). The combination of anlotinib and gemcitabine significantly inhibited the growth of ICC cells and induced cell apoptosis (Fan et al., 2021). In addition, anlotinib inhibited the proliferation, migration and invasion of BRCA cells and induce cell apoptosis by down-regulating TFAP2C (Fang and Yuan, 2021). Our results demonstrated that anlotinib significantly inhibited the proliferation of breast cancer cells in a time- and dose-dependent manner. The combination of anlotinib with DDP showed enhanced efficacy in inhibiting breast cancer cell proliferation and metastasis. Therefore, the combination therapy is anticipated to be a novel strategy for the treatment of BRCA.

Apoptosis and autophagy, representing type I and type II programmed cell death, respectively, are closely associated with the occurrence and progression of cancer (Deng et al., 2020; Abdullah et al., 2021; Wu et al., 2023). Therefore, we further discovered the effects of anlotinib combined with DDP on the apoptosis and autophagy of BRCA cells. Anlotinib significantly induced apoptosis in BRCA cells, and notably, when combined with DDP, it enhanced the expression of pro-apoptotic proteins while decreasing the expression of anti-apoptotic proteins in BRCA cells. Similarly, Ruan et al. (2019) found that anlotinib exhibited an antitumor effect on THCA cells by inhibiting cell growth, migration and inducing cell apoptosis. Another study showed that anlotinib significantly inhibited the proliferation of hepatocellular carcinoma (HCC) cells (He et al., 2018). Study also showed that anlotinib induced the autophagy in colon cancer cells (Sun et al., 2020). In this study, we found that anlotinib decreased P62 protein and enhanced the LC3-B, thus inducing autophagy in BRCA cells. LC3 and P62 were key proteins in autophagy, playing crucial roles in the formation and clearance of autophagosomes (Yorimitsu and Klionsky, 2005; Mizushima and Yoshimori, 2007; Lane et al., 2017).

It had been reported that JAK2/SATAT3 pathway was closely related to apoptosis and autophagy (Zhang et al., 2020; Zhong et al., 2021). In NSCLC and glioblastoma, anlotinib played an anticancer role by inducing apoptosis and autophagy through the JAK2/STAT3 pathway (Liang et al., 2019; Xu et al., 2022). In this study, we observed that anlotinib inhibited the phosphorylation levels of VEGFR2, JAK2, and STAT3, but could not the total protein of JAK2 and STAT3 (Figure 8F).

There are several limitations to our study. Firstly, we used a single 5 mg/kg dose of anlotinib for *in vivo* experiments, based on its previously demonstrated efficacy in breast cancer xenograft models. While this dose balanced effectiveness and low toxicity, using only one dose limited our understanding of dose-response relationships and potential dose-dependent effects. Future studies still need to explore a range of anlotinib doses to better characterize its pharmacodynamics, therapeutic window, and optimal dosing strategies for clinical translation. Secondly, our study demonstrates the synergistic effects of anlotinib and DDP *in vitro*; however, the *in vivo* experiments were limited to the effects of anlotinib alone. This limitation prevents a comprehensive characterization of the efficacy and safety of the combination therapy in a physiological context. Although logistical constraints precluded additional animal studies at this stage, our promising *in vitro* findings indicate that the combination may lead to enhanced tumor inhibition. In future study, we will focus on evaluating the anlotinib-DDP combination in animal models to confirm the synergistic effects observed *in vitro* and explore the underlying molecular mechanisms *in vivo*. Such studies would provide a robust foundation for assessing the translational potential of this therapeutic regimen. Thirdly, while our study provides valuable insights into the effects of anlotinib and DDP through bioinformatics analysis, *in vitro* experiments, and mouse xenograft models, we acknowledge the limitation of not validating our findings in clinical samples. To increase the generalizability and clinical relevance of our results, future studies should validate our findings using patient-derived samples. This would provide stronger evidence for the therapeutic potential of anlotinib, alone or in combination with DDP, and enable the identification of biomarkers, optimization of dosing strategies, and assessment of safety and efficacy in a clinically relevant setting.

## 5 Conclusion

In conclusion, this study provides compelling evidence for the efficacy of anlotinib in the treatment of BRCA, both as a standalone therapy and in combination with DDP. Our findings also elucidate the mechanism behind anlotinib’s effectiveness, highlighting its role in inhibiting the JAK2/STAT3 pathway, a key regulator in cancer progression. These findings pave the way for further clinical exploration and development of anlotinib-based therapies, offering hope for improved outcomes in breast cancer management.

## Data Availability

The original contributions presented in the study are included in the article/Supplementary Material, further inquiries can be directed to the corresponding authors.
