# Supplementary figures and images for: Synergistic effects of anlotinib and DDP on breast cancer: targeting the VEGF/JAK2/STAT3 axis
Source: Front Pharmacol. 2024 Oct 23;15:1494265. doi: 10.3389/fphar.2024.1494265 (PMC11537858; doi:10.3389/fphar.2024.1494265)

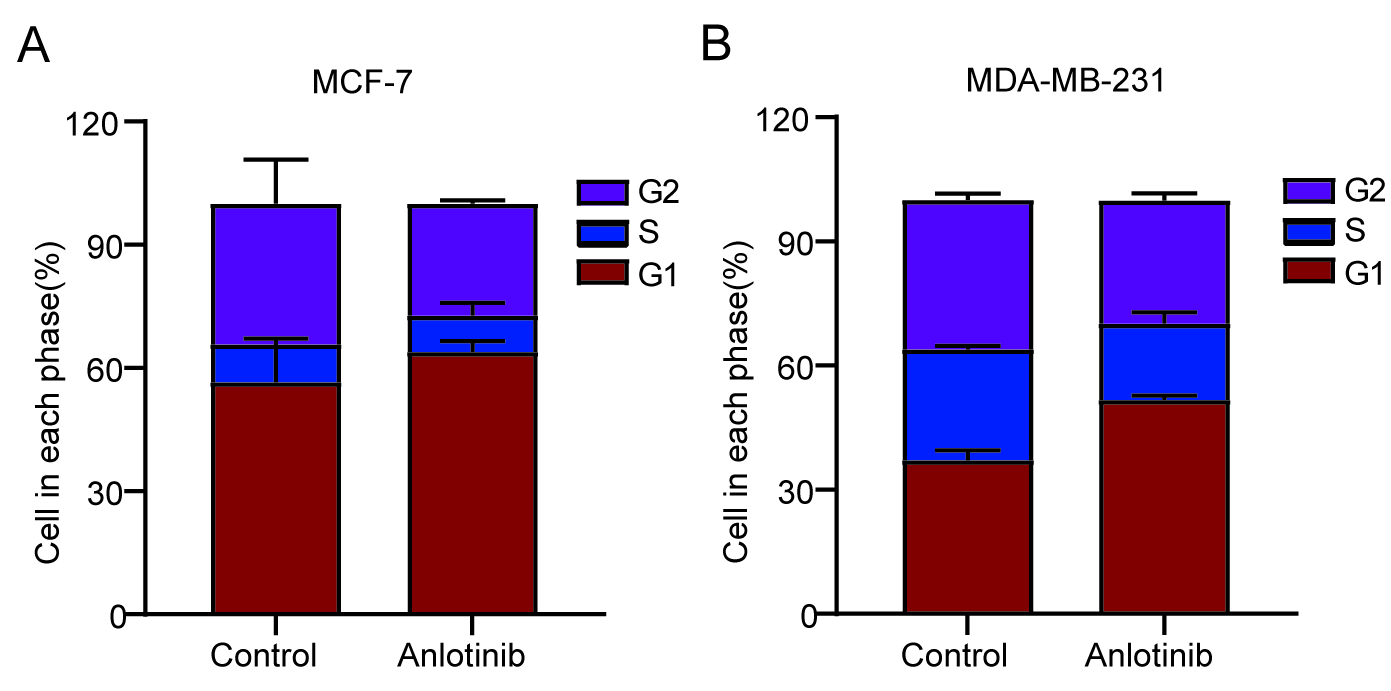

Supplement: Supplementary file 1 [file Image1.TIF]
