# Supplementary material for: Synergistic effects of anlotinib and DDP on breast cancer: targeting the VEGF/JAK2/STAT3 axis
Source: Front Pharmacol. 2024 Oct 23;15:1494265. doi: 10.3389/fphar.2024.1494265 (PMC11537858; doi:10.3389/fphar.2024.1494265)

The pictures below are the original MCF-7 cell line western blot results. The sample lanes are respectively control group, anlotinib group, cisplatin group, and anlotinib combined with cisplatin treatment group.

LC3B and P62:

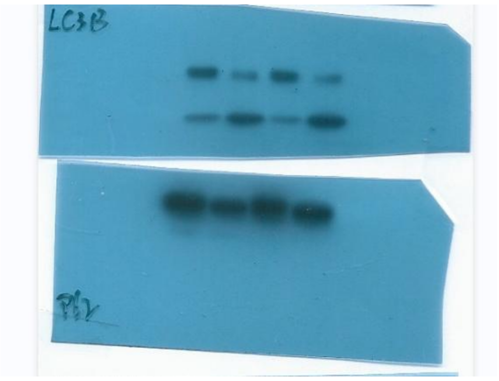

PARP1：

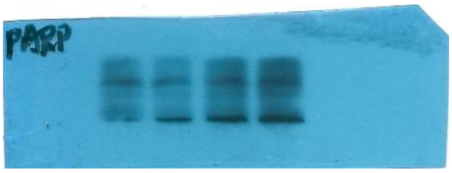

BAX:

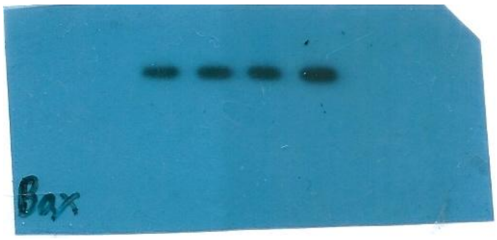

BCL2:

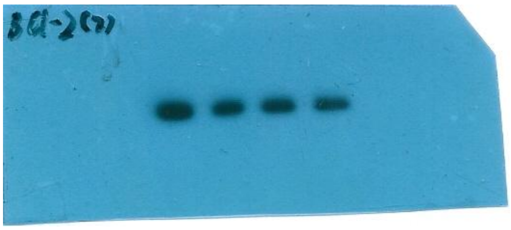

β-actin:

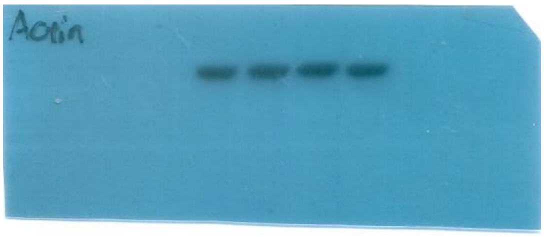

p-VEGFR2:

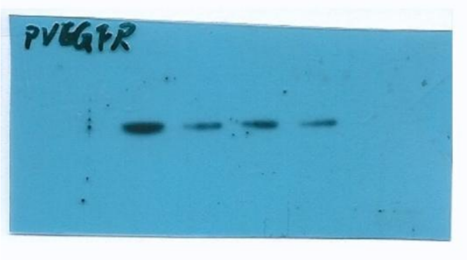

JAK2:

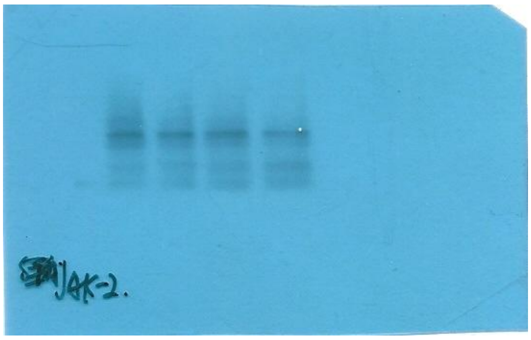

p-JAK2:

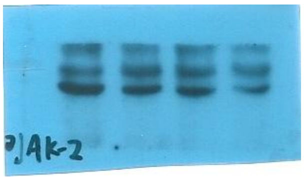

STAT3:

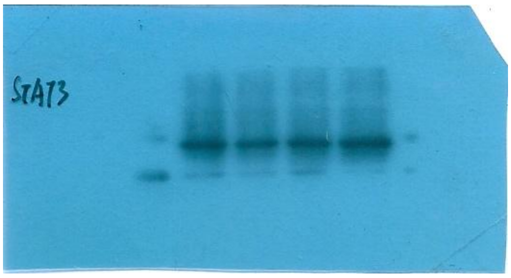

p-STAT3:

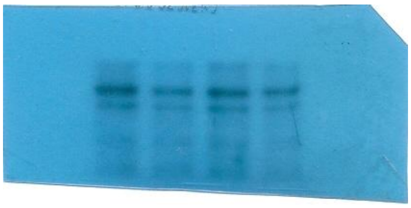

β-actin:

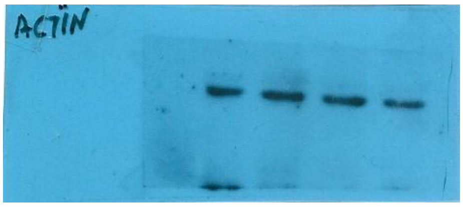

The pictures below are the original MDA-MB-231 cell line western blot results. The sample lanes are respectively control group, anlotinib group, cisplatin group, and anlotinib combined with cisplatin treatment group.

P62:

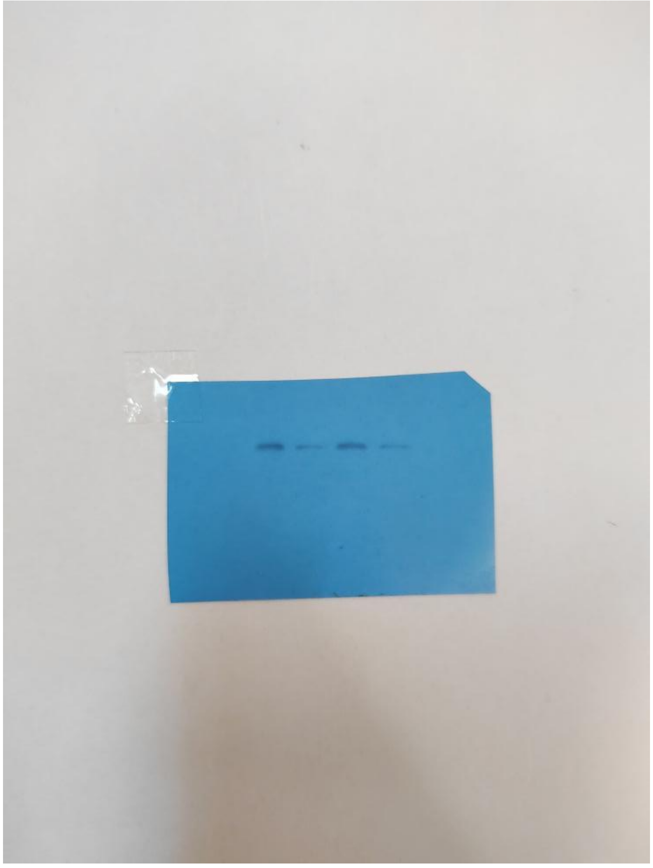

LC3B:

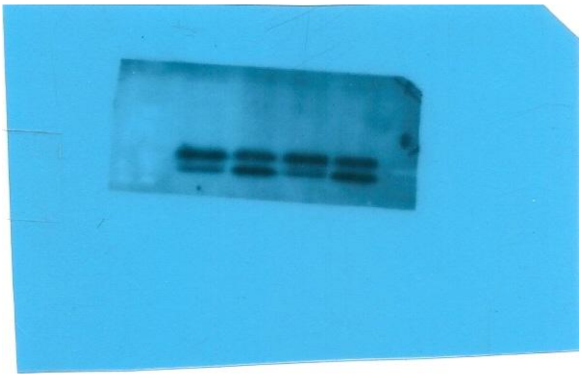

PARP1:

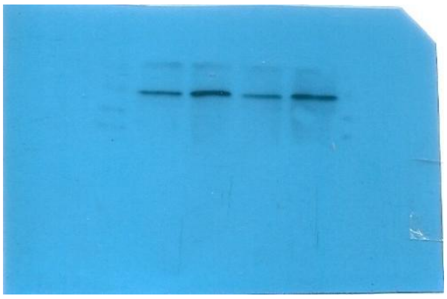

BAX:

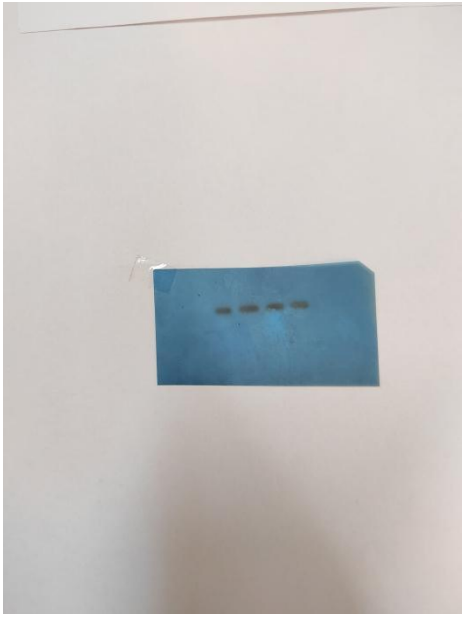

BCL2:

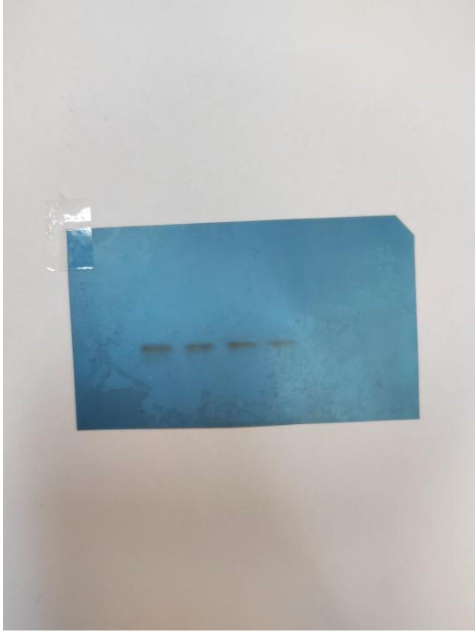

β-actin:

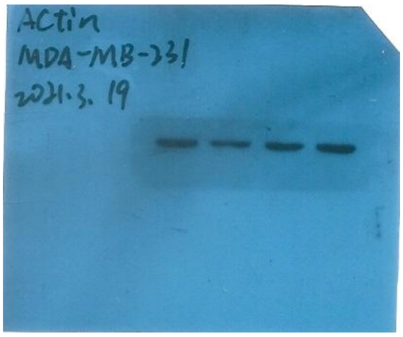

p-VEGFR2:

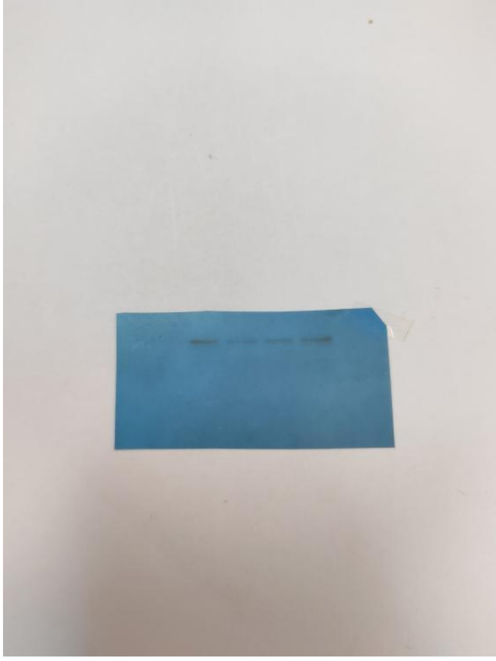

JAK2:

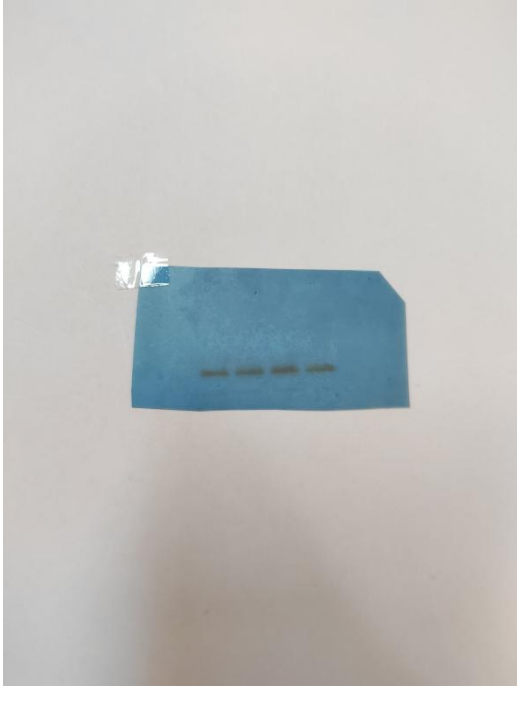

p-JAK2:

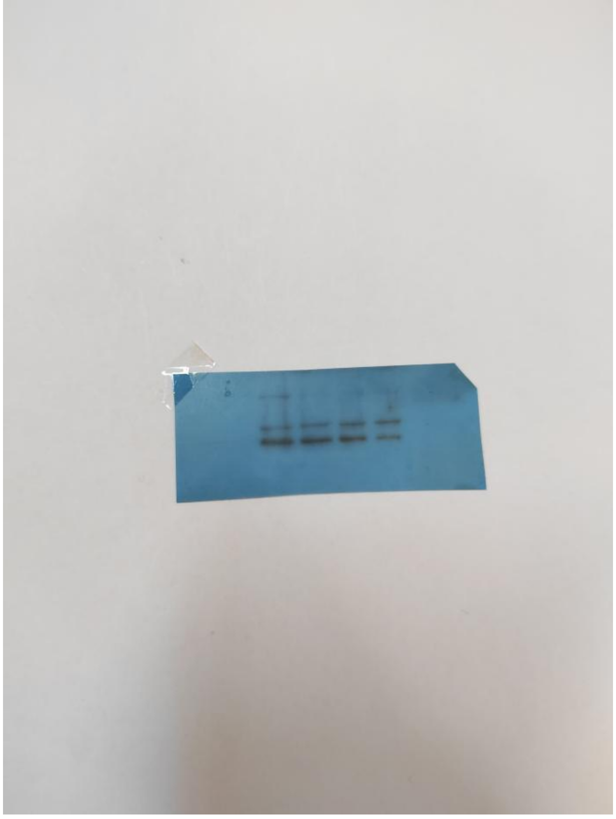

STAT3:

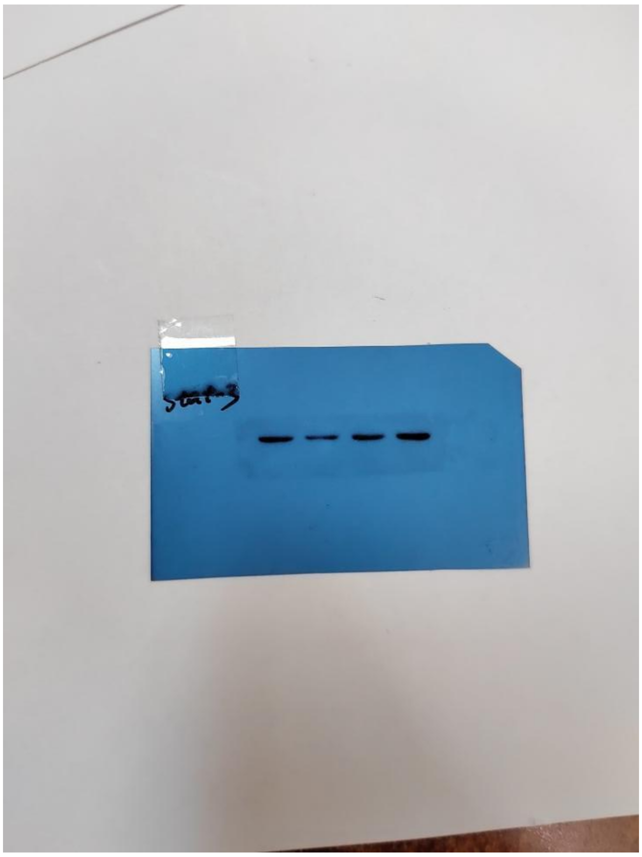

p-STAT3:

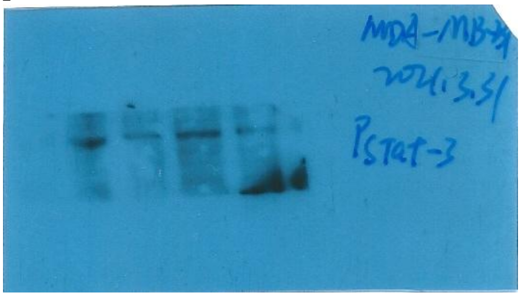

β-actin:

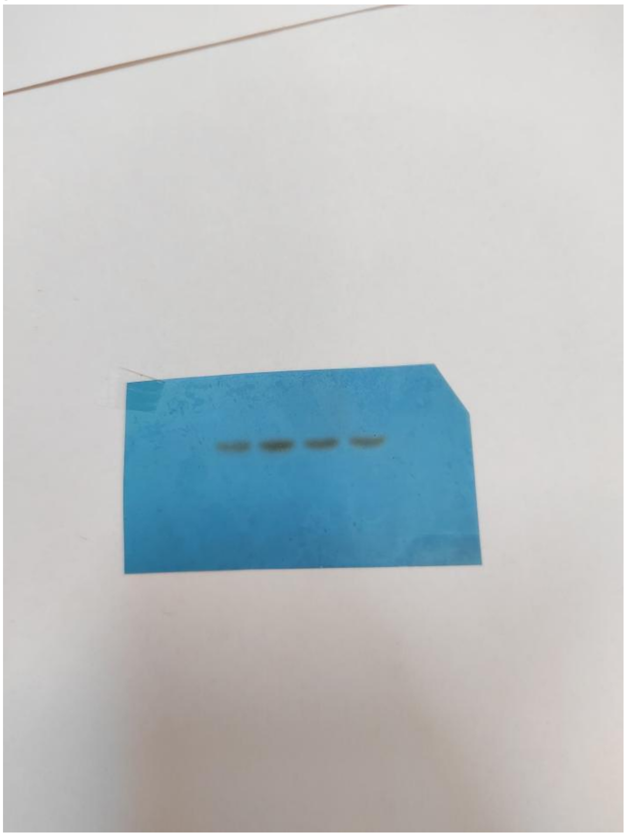

Supplement: Supplementary file 2 [file DataSheet1.PDF]
